# circCDYL2, Overexpressed in Highly Migratory Colorectal Cancer Cells, Promotes Migration by Binding to Ezrin

**DOI:** 10.3389/fonc.2021.716073

**Published:** 2021-08-17

**Authors:** Xiaomin Li, Jianjun Wang, Huaicheng Long, Weihao Lin, Haowei Wang, Yujia Chen, Qinzi Yuan, Xuenong Li

**Affiliations:** ^1^Guangdong Provincial Key Laboratory of Molecular Tumor Pathology, Department of Pathology, School of Basic Medical Sciences, Southern Medical University, Guangzhou, China; ^2^Department of Histology and Embryology, Wannan Medical College, Wuhu, China

**Keywords:** colorectal cancer, circular RNA, circCDYL2, hsa_circ_0004087, Ezrin

## Abstract

**Background:**

Colorectal cancer (CRC) is one of the most common malignancies with high mortality worldwide, particularly due to metastasis. However, there are no clinically available strategies for treating CRC metastasis. Exploring the mechanisms underlying CRC metastasis is the key to improve the treatment of CRC with metastasis.

**Methods:**

In this study, we generated the highly migratory CRC cell subline H-RKO using a repeated transwell migration assay to identify circRNAs involved in CRC migration by high-throughput RNA sequencing. Upregulated circRNAs were validated by RT-qPCR to identify the most elevated circRNA. The expression of this circRNA (circCDYL2) was evaluated in 40 pairs of CRC tissues and four CRC cell lines by RT-qPCR. Transwell migration and wound healing assays were performed to verify the function of circCDYL2 in cell migration. The cellular distribution of circCDYL2 was confirmed using PCR. RNA pulldown and RNA immunoprecipitation were used to confirm the interaction between circCDYL2 and Ezrin. Western blotting, immunohistochemistry, and rescue experiments were used to determine the role of circCDYL2 in regulating Ezrin protein expression and AKT phosphorylation.

**Results:**

Among the candidate circRNAs, circCDYL2 was the highest overexpressed circRNA in H-RKO compared to parental N-RKO cells. Furthermore, circCDYL2 expression was elevated in CRC tissues and cell lines. Gain- and loss-of-function assays indicated that circCDYL2 enhanced the migration of CRC cells. circCDYL2 was located in the cytoplasm of CRC cells and interacted with Ezrin to upregulate its protein levels, resulting in AKT phosphorylation. Ezrin knockdown abrogated the CRC cell migration induced by circCDYL2 overexpression.

**Conclusions:**

Our study demonstrated for the first time that circCDYL2 promotes CRC migration by binding Ezrin and activating the AKT pathway. CircCDYL2 represents a potential therapeutic target for preventing CRC metastasis.

## Introduction

Colorectal cancer (CRC) is one of the most common malignancies. According to the latest Global Cancer Statistics (2020), its incidence ranks third for males and second for females among all cancers, and it is the second cause of cancer death for both sexes combined (1). Metastasis contributes to the majority of CRC-related deaths. However, there are currently no therapeutic strategies for avoiding CRC metastasis. Therefore, it is important to investigate the underlying mechanisms of CRC metastasis to identify potential therapeutic targets and facilitate the development of preventive therapies, which are crucial for improving the CRC patient survival rate.

Most sequences of the human genome are transcribed into non-coding RNAs (ncRNAs), including microRNAs (miRNAs), long non-coding RNAs (lncRNAs), and circular RNAs (circRNAs). CircRNAs are of great interest, and their roles and mechanisms in tumorigenesis and progression are being investigated. Unlike linear RNA, circRNA forms a closed loop by back splicing its 5’ end and 3’ end terminals (2). This special structure makes circRNAs more stable and ideal markers or targets for cancer diagnosis and therapy.

Increasing evidence has revealed that dysregulation of circRNAs plays a crucial role in tumor growth, invasion, metastasis, stemness, angiogenesis, immune escape, and drug resistance in breast cancer, hepatocellular carcinoma, glioma, and CRC (3–5). The predominant function of circRNAs is as “miRNA sponges” to regulate miRNA targets by sequestering the miRNAs. CircRNAs also bind to proteins (6) and encode proteins and peptides (7, 8). Several circRNAs have been reported to influence CRC metastasis (9–12), indicating that circRNAs may represent therapeutic targets against CRC metastasis. However, metastasis-associated circRNAs and their roles and mechanisms in this disease process remain poorly defined in CRC.

In this study, we generated the highly migratory CRC cell subtype H-RKO from the RKO CRC cell line (N-RKO) using a repeated transwell migration assay and performed high-throughput RNA sequencing to identify overexpressed circRNAs that could be involved in CRC metastasis. We discovered a novel circRNA, circCDYL2, that was overexpressed in H-RKO, CRC tissues, and CRC cell lines. Functional assays showed that circCDYL2 promoted CRC cell migration. Furthermore, circCDYL2 physically interacted with the Ezrin protein to upregulate its protein expression and promote AKT phosphorylation, enforcing CRC cell migration. Thus, circCDYL2 could represent a potential preventive and therapeutic target for the treatment of CRC.

## Materials and Methods

### Cell Culture

The normal colonic epithelial cell line FHC and the CRC cell lines SW620, RKO, SW480, and HCT116 were purchased from the American Type Culture Collection (ATCC, Manassas, VA, USA). Cells were cultivated in RPMI 1640 medium for CRC cell lines or DMEM medium for FHC (#C11875500BT, #C11995500BT, Gibco, Grand Island, NY, USA) supplemented with 10% or 20% fetal bovine serum (FBS) at 37°C with 5% CO_2_.

### CRC Tissue Samples

CRC tissues and the matched adjacent non-cancerous colorectal mucosal tissues were collected from the general surgically resected CRC specimens at Nanfang Hospital, Southern Medical University (Guangzhou, China), with consent from all patients. The use of clinical materials for research purposes were approved by the Ethics Committee of Southern Medical University (Guangzhou, China). CRC tissues were taken from the CRC focus and avoid the obvious necrosis, while the adjacent non-cancerous tissues were taken from the colorectal mucosal tissues 5–10 cm away from the edge of the tumors. These fresh dissected samples were immediately frozen in liquid nitrogen until further use. Forty CRC samples were involved in the current study, namely, 22 men and 18 women. The age ranges from 28 to 88 years, and the median age is 60 (20 cases ≥ 60, 20 cases < 60). All samples were diagnosed to be colorectal adenocarcinoma, and their matched adjacent tissues were proven to have no cancer cells through pathological test. All patients did not undergo chemotherapy, radiotherapy, or other targeted therapy before surgery.

### RNA Sequencing

RNA sequencing was carried out in Riobio tech (Guangzhou, China). Briefly, total RNA was extracted from H-RKO and N-RKO cells using the RNAiso Plus reagent (#9109, Takara, Japan) according to the manufacturer’s protocol. RNA purity was evaluated by ND-1000 Nanodrop with the requirement A260/280 ≥ 1.8, A260/230 ≥ 2.0. RNA integrity (RIN) was assessed with requiring RIN ≥ 7.0. Then, rRNAs were removed using QIAGEN QIAseq FastSelect RNA Removal Kit (334387, QIAGEN, Germany), and RNA was treated with RNase R (#RNR07250, Epicentre, USA) and fragmented to approximately 200 bp. Subsequently, the purified RNA fragments were subjected to first-strand and second-strand cDNA synthesis followed by adaptor ligation and enrichment with a low cycle according to instructions of NEBNext^®^ Ultra™ RNA Library Prep Kit for Illumina (#E7530L, NEB, USA). The purified library products were sequenced on HiSeq X Ten mode. Two algorithms, CIRI2 and CIRCexplorer2, were used to detect circRNAs. If a circRNA could be detected by both methods, it would be considered as an identified circRNA. The RNA-seq raw data have been deposited in the NCBI Sequence Read Archive (SRA) database: https://www.ncbi.nlm.nih.gov/sra/PRJNA732514. The BioProject accession number is PRJNA732514.

### RNA Extraction, Reverse Transcription, Sanger Sequencing, PCR, and Quantitative Real-time PCR (RT-qPCR)

Total RNAs were extracted with RNAiso Plus reagent (#9109, Takara, Japan). PrimeScriptTM RT Reagent Kit with gDNA Eraser (#RR047A, Takara, Japan) was used to conduct reverse transcription. Sanger sequencing was carried out at Ruibiotech (Guangzhou, China). PCR reactions were performed using PrimeSTAR GXL DNA Polymerase (#R050Q, Takara, Japan) according to the manufacturer’s instructions. RT-qPCR was performed to measure RNA expression using SybrGreen qPCR Mastermix (#DBI-2043, DBI Bioscience, Germany) in the ABI 7500/7500 Fast Real-Time PCR System (Applied Biosystems, USA). GAPDH was used as an internal control. The RT-qPCR data were analyzed using the comparative C(T) method (13). In brief, each sample’s threshold cycle value (Ct) of target was generated by performing RT-qPCR, and then the relative expression of circCDYL2 was calculated by normalizing to the internal control gene GAPDH and shown as −ΔCt value (−ΔCt = Ct _GAPDH_ − Ct _circCDYL2_) or 2^−ΔCt^ value (2^−ΔCt^ = 2^Ct GAPDH − Ct circCDYL2Ct^). All primers used in PCR and RT-qPCR were synthesized by Ruibiotech (Guangzhou, China), and the sequences are listed in **Supplementary Table 1**.

### Plasmid, siRNAs, and Transfection

The circCDYL2 expression vector was constructed at Ruibiotech (Guangzhou, China) using the circRNA expression plasmid pCD5-ciR (#GS0105, Geneseed Biotech, Guangzhou, China). All small interfering RNAs (siRNAs) used in this study were synthesized by Riobio tech (Guangzhou, China). The siRNA-targeted sequences are listed in **Supplementary Table 2**. Plasmid and siRNA transfection was performed using Lipofectamine 3000 (#L3000015, Invitrogen, USA) according to the manufacturer’s protocol.

### Transwell Migration Assay

Cells (0.5 × 10^5^ or 1 × 10^5^) were suspended in 300 μl of serum-free medium. Then, these cell suspensions were added into transwell chambers (#353097, BD Falcon, USA) and 700 μl of medium containing 20% FBS was put into the plate. After 48 h, the migratory cells were stained with Giemsa (#KGA227, KeyGen Biotech, China) according to the manufacturer’s instruction. Every chamber was captured in five fields (×200) under a microscope and counted to quantify the migratory cells.

### Wound Healing Assay

Cells were seeded in six-well plates and then treated with vector or siRNA targeting circCDYL2 or Ezrin and incubated for roughly 48 h to make the cells reach approximately 90% confluence. A 10-μl plastic pipette tip was used to produce the scratch in each well. After the scratch, wounds were monitored and photographed in five fields (×100) at 0 h, 24 h, and 48 h. The migratory abilities of cells were assessed by measuring the migratory distance and calculating the migration rate.

### Separation of Cytoplasm and Nucleus Fraction

The PARIS Kit (#AM1921, Life Technologies, Austin, TX, USA) was used to separate cytoplasmic and nuclear RNAs of cells according to the manufacturer’s instruction. Briefly, cells were collected and lysed to achieve the cytoplasm fraction. Then, the same volume lysed buffer was used to lyse the nuclear precipitation and thus obtain the nucleus fraction. The cytoplasmic and nuclear RNAs were extracted and subjected to RT and PCR with primers of circCDYL2, GAPDH, and U6, respectively.

### RNA Pulldown

Firstly, circCDYL2 and its antisense RNA were synthesized with RNA polymerase using an *in vitro* transcription method according to the instruction of the MEGAscript Kit (#AM1333, Life, USA), followed by biotinylation labeling reactions using the Pierce RNA 3′ End Desthiobiotinylation Kit (#20163, Thermo, USA). Then, RNA pulldown was carried out according to the procedure of Pierce Magnetic RNA-Protein Pull-Down Kit (#20164, Thermo, USA). Briefly, a total of 50 pmol labeled RNAs were captured by 50-µl streptavidin magnetic beads, mixed with protein lysate, and incubated for 1 h at 4°C with rotation. Washing and elution of RNA-binding protein complexes was conducted. The eluted proteins were separated by SDS-PAGE followed by silver staining (#P00175, Fast Silver Stain Kit, Beyotime, China). Then, the silver-stained differential protein band in gel was cut off and subjected to digestion with trypsin at 37°C overnight. The enzyme-digested polypeptide samples were dried and dissolved again for mass spectrometry analysis (Wininnovate Bio, Shenzhen, China).

### RNA Immunoprecipitation (RIP)

RIP assay was performed using 5 µg of Ezrin antibody (#26056-1-AP, proteintech, USA) or Rabbit Control IgG (#AC005, ABclonal, China) with RNA Immunoprecipitation Kit (#P0101, Geneseed Biotech, Guangzhou, China). In brief, cells were collected and lysed on ice using Buffer A provided by the kit. Magnetic beads (100 µl) and about 5 µg of antibody were incubated at 4°C for 2 h to form a magnetic beads–antibody complex. Then, the complexes and cell lysate were incubated at 4°C for 2 h. After washing, RNA was purified with RC Columns and the protein was extracted using acetone and ethyl alcohol. RT-qPCR and PCR were used to detect circCDYL2 enrichment, while Western blot assays were used to confirm the enrichment of Ezrin protein.

### Western Blot

CRC cells were lysed on ice by lysis buffer (#KGP701, KeyGen Biotech, China) followed by quantificational measure with a bicinchoninic acid (BCA) Protein Assay Kit (#KGPBCA, KeyGen Biotech, China). Then, the lysates were subjected to SDS-polyacrylamide gel electrophoresis for separation. The isolated proteins were transferred onto polyvinylidene fluoride (PVDF) membrane (#IPVH00010, Millipore, USA). After fixing in methanol for 1 min, the PVDF membranes were blocked in PBST solution containing 5% nonfat milk and subsequently incubated overnight at 4°C with the following primary antibodies, respectively: anti-Akt (1:1,000 dilution, #YT0178, immunoway, USA), anti-p-Akt (Ser-473) (1:1,000, #YP0006, immunoway, USA), anti-Ezrin (1:5,000 dilution, #26056-1-AP, proteintech, USA), and anti-β-tubulin (1:5,000 dilution, #10094-1-AP, proteintech, USA). Then, the PVDF membranes were incubated with the secondary antibodies (Goat anti-rabbit IgG, 1:5,000 dilution, #BS13278, Bioworld, USA; Goat anti-mouse IgG, 1:5,000 dilution, #BS12478, Bioworld, USA) and were visualized with FDbio-Dura ECL Kit (#FD8020, Fdbio, China). All experiments were repeated three times and quantified using Gel-pro software.

### Immunohistochemistry (IHC)

The 4-µm-thick CRC paraffin sections were deparaffinized in xylene, rehydrated through decreasing concentrations of ethanol, and washed in PBS. Then, the slides were heated in 0.01 M, pH 6.0 citrate buffer for about 5 min to unmask antigens and incubated in 3% H_2_O_2_ to inhibit endogenous peroxidase activity. After antigen retrieval, the slides were incubated with anti-p-Akt antibody (Ser-473) (1:200, #YP0006, immunoway, USA) overnight at 4°C, followed by incubation with the secondary antibody (#PV-6001, ZSGB-BIO, China) and visualization with DAB color development kit (#ZLI-9018, ZSGB-BIO, China). The expression of p-AKT in each tumor sample was quantified using H-score (H-score = % of weak staining × 1 + % of moderate staining × 2 + % of strong staining × 3), which ranges from 0 to 300 (14).

### Statistical Analysis

Statistical analyses were performed using the GraphPad Prism version 6.0 (GraphPad Software, La Jolla, CA, USA). Data were presented as mean ± standard deviation. Pearson correlation coefficient was performed to check the correlation between circCDYL2 expression and p-AKT levels in CRC tissues. Differential analysis between groups were achieved with the two-tailed Student’s *t*-test. The bands of Western blots were analyzed by the Gel-pro analyzer software (Media Cybernetics, Rockville, MD, USA). Differences were considered to be significant when *p* < 0.05.

## Results

### Generation of a Highly Migratory CRC Cell Subline From the RKO Cell Line

**Figure 1:**
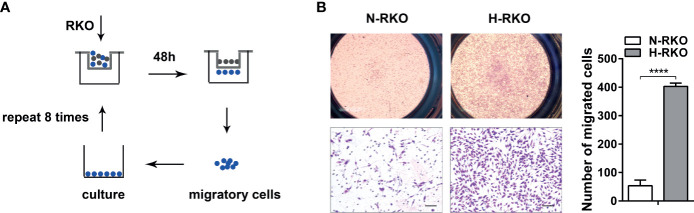
Generation of a highly migratory CRC cell subline from the RKO cell line. **(A)** Procedure summary schematic for establishing a CRC cell subline with a high migratory potential from parental RKO cells (N-RKO) using a repeated transwell assay. H-RKO: the highly migratory RKO; N-RKO: the parental RKO. **(B)** Transwell assay showed that H-RKO had a higher migration rate compared to the parental N-RKO cells. Top: 40 ×; bottom: 200 ×. Scale bars: 50 μm. ****p < 0.0001.

We generated a CRC cell subline with a high migratory potential from parental RKO cells (N-RKO) using a repeated transwell assay. First, an N-RKO suspension was seeded in the inner transwell chamber. After 48 h, the migratory cells from the membrane of the chamber were collected after trypsin digestion and cultured in a new plate. This process was repeated eight times. The highly migratory subtype H-RKO was established after the eighth round (**Figure 1A**). Cell migration was reassessed using the transwell migration assay. We found that subline H-RKO had a higher migration rate compared to the parental N-RKO cells (**Figure 1B**).

### CircCDYL2 Is Overexpressed in H-RKO

**Figure 2:**
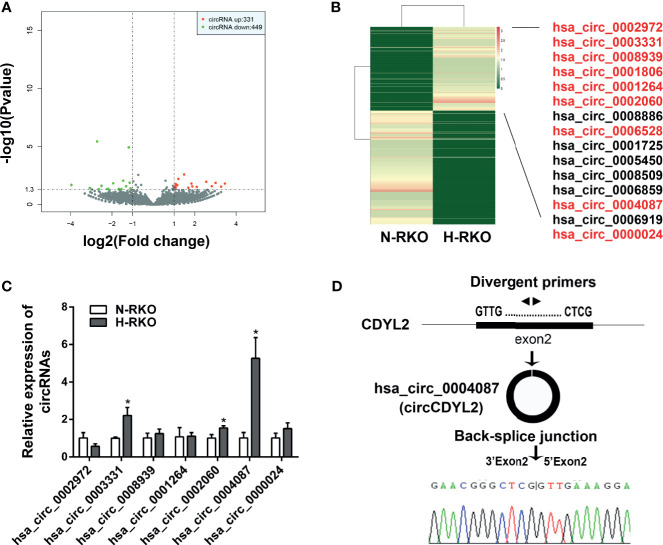
CircCDYL2 was overexpressed in H-RKO. **(A)** Volcano plot of the differentially expressed circRNAs in H-RKO compared with N-RKO. Red: upregulated circRNAs. Green: downregulated circRNAs. **(B)** Heatmap of the differentially expressed circRNAs in H-RKO compared to N-RKO. Of the upregulated circRNAs, 15 were expressed in both H-RKO and N-RKO and 9 circRNAs with a length of less than 1000 bp marked with red color. **(C)** The relative expression levels of seven upregulated circRNAs in H-RKO were reassessed using RT-qPCR. **(D)** Schematic shows that circCDYL2 originated from exon 2 of CDYL2 gene by back-splicing. The divergent primers were designed to detect circCDYL2. Sanger sequencing confirmed that the back-splicing junction sequences of circCDYL2 were consistent with the head-to-tail sequences of CDYL2 exon 2. *p < 0.05.

To find the circRNAs involved in the initiation of migration, we performed high-throughput RNA sequencing. Using *p* < 0.05 and |fold change| > 2, we identified 331 upregulated circRNAs and 449 downregulated circRNAs in the H-RKO cells compared to N-RKO (**Figure 2A** and **Supplementary Table 3**). Of the upregulated circRNAs, 15 were expressed in both H-RKO and N-RKO (**Figure 2B**). To filter out the target circRNAs, we reassessed the expression of 9 circRNAs with a length less than 1000 bp using RT-qPCR. This analysis excluded hsa_circ_0006528 for its low expression and hsa_circ_0001806, whose function and underlying mechanism in CRC were reported previously (15). Among the remaining seven circRNAs, hsa_circ_0004087 was the most elevated in H-RKO cells (**Figure 2C**). According to circBase (16), this circRNA originated from exon 2 of the chromodomain Y like 2 (CDYL2) gene, with 592 bp spliced length. Thus, we named hsa_circ_0004087 circCDYL2 hereafter. Sanger sequencing confirmed that the back-splicing junction sequences of circCDYL2 were consistent with the head-to-tail sequences of CDYL2 exon 2 (**Figure 2D**).

### CircCDYL2 Is Upregulated in CRC Tissues and Cell Lines

**Figure 3:**
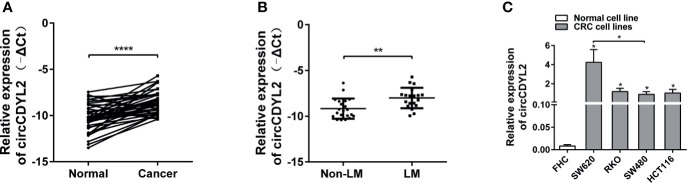
CircCDYL2 was upregulated in CRC tissues and cell lines. **(A)** The relative expression levels of circCDYL2 were detected in 40 paired CRC tissues (Cancer) and matched adjacent non-cancerous colorectal mucosal tissues (Normal) by RT-qPCR. **(B)** CRC tissues with lymphatic metastasis (*n* = 19) had higher circCDYL2 expression levels than CRC tissues without lymphatic metastasis (*n* = 21). **(C)** CircCDYL2 was overexpressed in four CRC cell lines compared with the normal human colonic cell line FHC. *p < 0.05, **p < 0.01, ****p < 0.0001.

RT-qPCR was performed to measure the circCDYL2 expression levels in CRC tissues and cell lines. CircCDYL2 was significantly upregulated in 40 CRC tissues compared to the matched non-tumorous adjacent tissues (**Figure 3A**). Furthermore, CRC tissues with lymphatic metastasis (*n* = 19) had higher circCDYL2 expression levels than CRC tissues without lymphatic metastasis (*n* = 21) (**Figure 3B**). We also examined circCDYL2 expression levels in CRC cell lines and found that circCDYL2 was overexpressed in all four evaluated CRC cell lines compared with the normal human colonic cell line FHC. Moreover, its expression was higher in SW620, a cell line of metastatic origin, compared to SW480, a cell line of primary origin (**Figure 3C**). These data indicated that the increased circCDYL2 expression in CRC could be related to metastasis.

### CircCDYL2 Promotes CRC Cell Migration *In Vitro*

**Figure 4:**
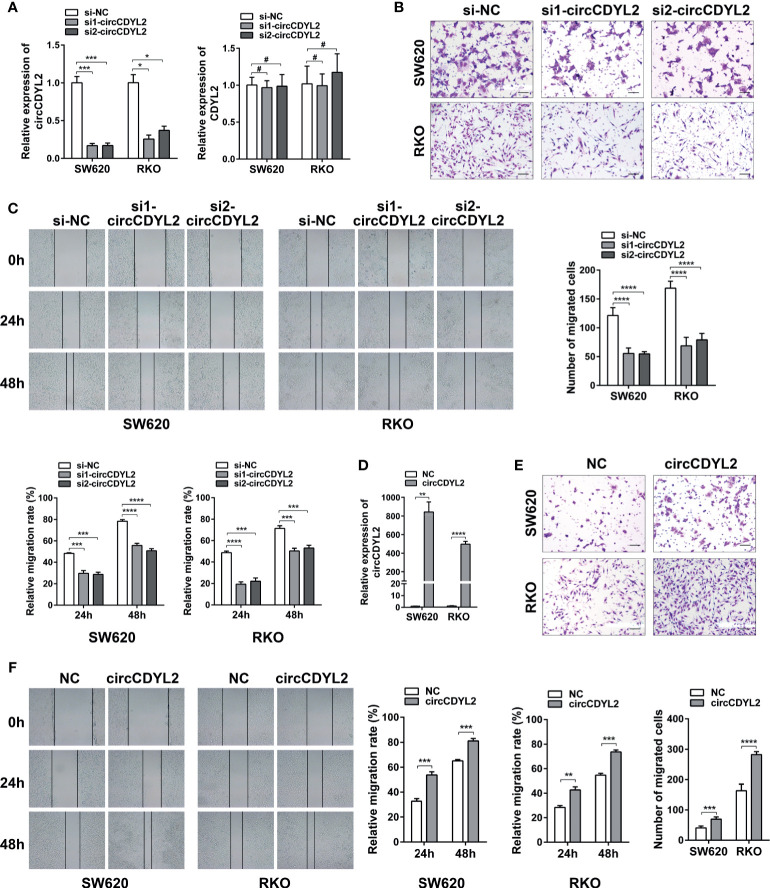
CircCDYL2 promoted CRC cell migration *in vitro*. **(A)** The relative expression levels of circCDYL2 and CDYL2 in SW620 and RKO after transfection with siRNAs targeting the circCDYL2 splicing junction. **(B)** Transwell migration assay demonstrated that decreased circCDYL2 levels suppressed CRC cell migration. Scale bars: 50 μm. **(C)** Wound healing assay indicated that silencing circCDYL2 inhibited CRC cell migration. **(D)** CircCDYL2 was overexpressed after transfection with circCDYL2 expression vector. **(E)** Transwell migration assay showed that ectopic circCDYL2 increased cell migration. Scale bars: 50 μm. **(F)** Wound healing assay indicated that overexpression of circCDYL2 enhanced CRC cell migration. *p < 0.05, **p < 0.01, ***p < 0.001, ****p < 0.0001, ^#^ *p* > 0.05.

To determine the roles of circCDYL2 in CRC metastasis, we conducted gain- and loss-of-function experiments. Using two independent siRNAs targeting the circCDYL2 splicing junction, we knocked down circCDYL2 expression in SW620 and RKO cells without affecting the expression of the CDYL2 parental gene (**Figure 4A**). Both the transwell migration and wound healing assays showed that decreased circCDYL2 levels inhibited CRC cell migration (**Figures 4B, C**). In contrast, overexpression of circCDYL2 in SW620 and RKO enhanced cell migration (**Figures 4D–F**). These findings confirmed that circCDYL2 could promote the migration of CRC cells.

### CircCDYL2 Physically Binds to Ezrin Protein

**Figure 5:**
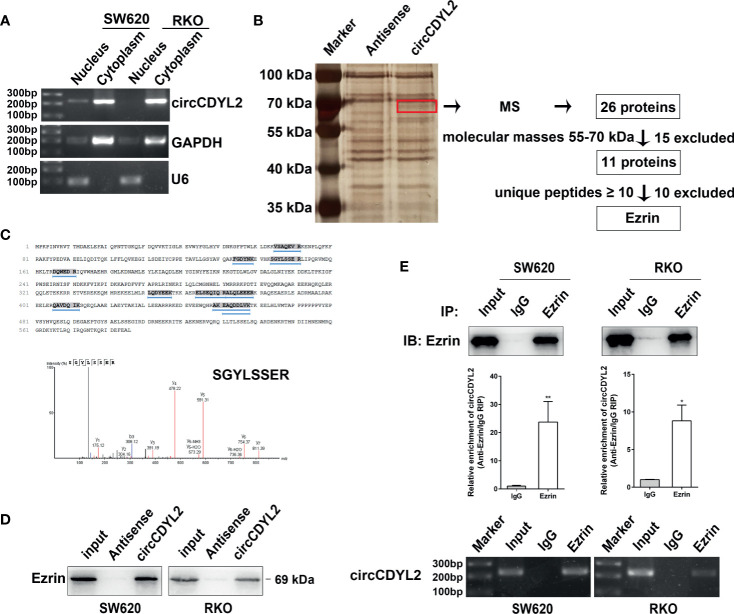
CircCDYL2 mainly resided in the cytoplasm of CRC cells and bound to Ezrin. **(A)** The nuclear and cytoplasmic distribution of circCDYL2 was confirmed using nucleic acid electrophoresis following PCR. GAPDH was mainly localized in cytoplasm while U6 was mainly localized in nucleus. They were used as the quality control of cell fractionation. **(B)** RNA pulldown assays were used to identify possible target proteins that circCDYL2 could bind to. After silver staining, a differential band marked by red frame was observed in the circCDYL2 group compared to the control. Twenty-six proteins were identified in this band by MS, and Ezrin was determined to be a candidate target protein of circCDYL2. **(C)** The amino acid sequences of Ezrin and 10 unique peptides identified by MS. Below is the peak diagram of peptide. **(D)** Western blotting following RNA pulldown confirmed the binding between circCDYL2 and Ezrin. **(E)** RT-qPCR and PCR showed enrichment of circCDYL2 following RIP with an Ezrin antibody. *p < 0.05, **p < 0.01.

To investigate the possible mechanism underlying the functions of circCDYL2 in CRC cell migration, we first evaluated the cellular localization of circCDYL2 because it is known that the functions of circRNAs are related to their subcellular distribution (17). We found that circCDYL2 mainly resided in the cytoplasm of CRC cells (**Figure 5A**).

Recently, studies revealed that circRNAs can interact with proteins (6, 18). We performed RNA pulldown assays in RKO cells to identify possible target proteins that circCDYL2 could bind to. We observed a differential band between 55 and 70 kDa in the circCDYL2 pulldown group compared to the control. Twenty-six proteins were identified in this band by mass spectrometry (MS) (**Supplementary Table 4**). Ezrin was determined to be a candidate target protein of circCDYL2 because its molecular mass was between 55 and 70 kDa, and it contained the most identified unique peptides (**Figures 5B, C**). Consistent with the MS findings, Western blotting following RNA pulldown confirmed the binding between circCDYL2 and Ezrin (**Figure 5D**). Furthermore, RT-qPCR and PCR showed enrichment of circCDYL2 following RIP with an Ezrin antibody (**Figure 5E**). These results demonstrated that circCDYL2 could interact with Ezrin.

### CircCDYL2 Promotes AKT Phosphorylation by Upregulating Ezrin Protein Levels

**Figure 6:**
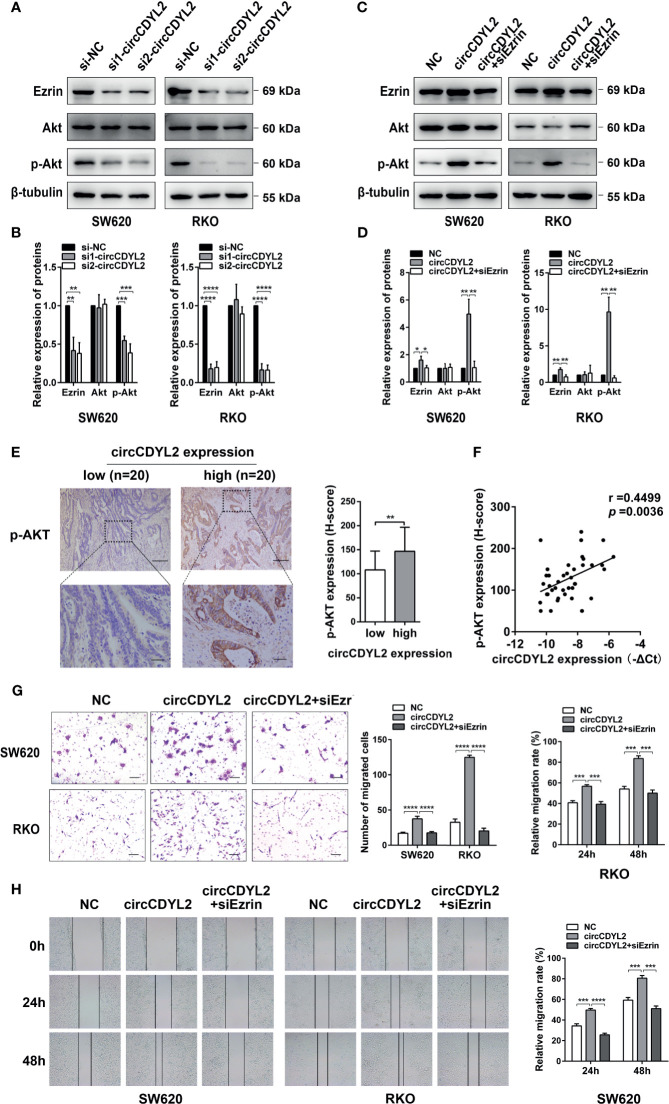
CircCDYL2 promoted AKT phosphorylation by upregulating Ezrin protein levels. **(A)** Western blotting revealed that silencing circCDYL2 expression resulted in decreases in Ezrin protein levels and Akt phosphorylation. **(B)** Quantitative graphs for 6A. **(C)** Western blotting showed that overexpression of circCDYL2 caused increased Ezrin protein expression and AKT phosphorylation. These effects could be abolished by co-transfection with Ezrin siRNA. **(D)** Quantitative graphs for 6C. **(E)** IHC showed that p-AKT levels in CRC tissues were higher in the high circCDYL2 group (*n* = 20) than in the low circCDYL2 group (*n* = 20). Scale bars: Top, 100 μm; bottom, 20 μm. **(F)** The correlation analysis demonstrated a positive correlation between circCDYL2 expression and p-AKT levels. *n* = 40. **(G)** Transwell and **(H)** wound healing assays showed that enhanced CRC cell migration caused by circCDYL2 overexpression could be abolished by Ezrin knockdown. *p < 0.05, **p < 0.01, ***p < 0.001, ****p < 0.0001.

Ezrin is a cytoskeletal organizer that promotes metastasis in many cancer types by reorganizing the cytoskeleton or controlling the signal transduction [e.g., activating NF-κB, epithelial–mesenchymal transformation (EMT), and AKT pathways] (19–22). Considering the crucial role of the AKT pathway in tumor progression, we examined the effect of circCDYL2 on Ezrin expression and AKT phosphorylation by Western blotting. Data revealed that silencing circCDYL2 expression resulted in decreases in Ezrin protein levels and Akt phosphorylation (**Figures 6A, B**). In contrast, overexpression of circCDYL2 caused increased Ezrin protein expression and AKT phosphorylation. These effects could be abolished by co-transfection with Ezrin siRNA (**Figures 6C, D**).

**Figure 7:**
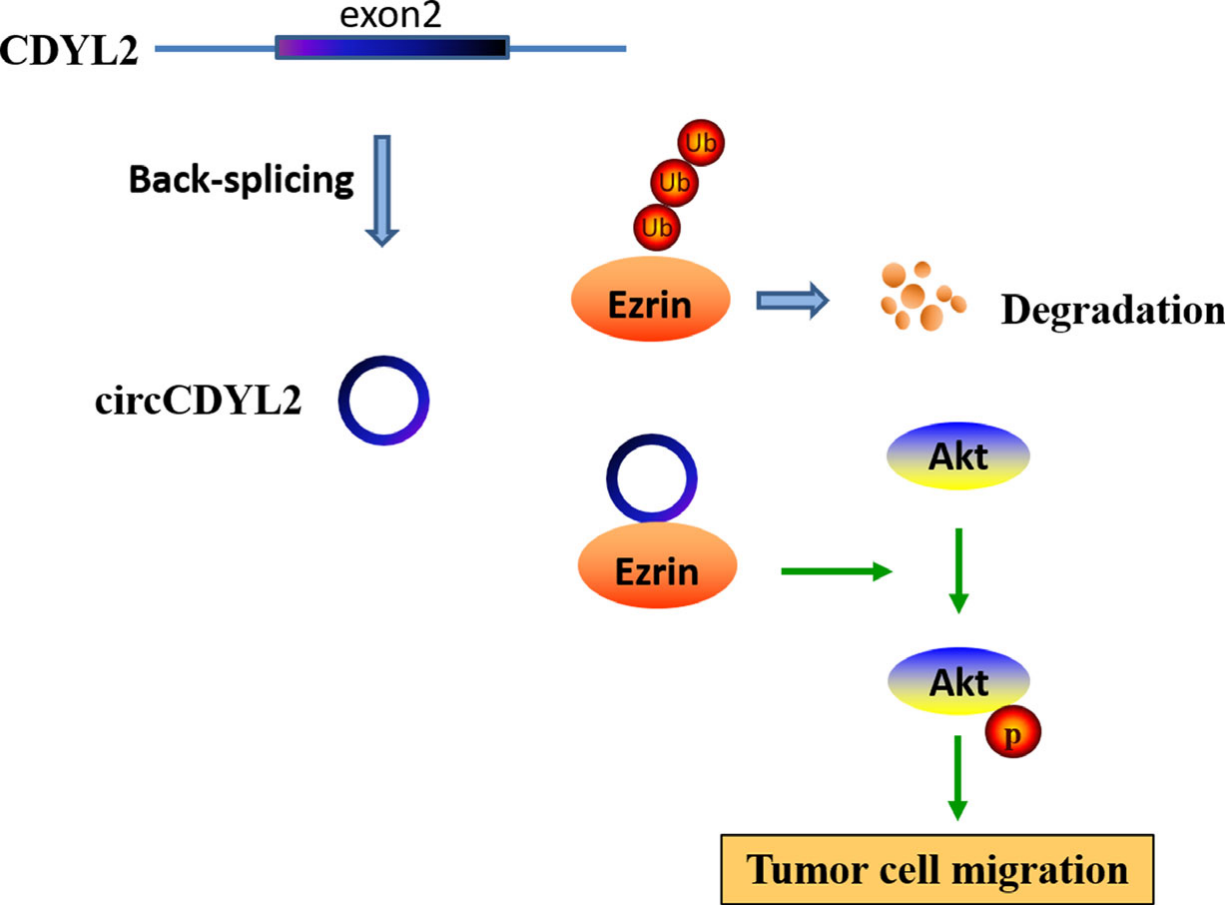
A hypothesis model of circCDYL2 involvement in CRC migration.

To further investigate the regulatory role of circCDYL2 in AKT phosphorylation, we performed IHC to determine p-AKT levels in the 40 CRC tissues used in **Figure 3A**. Moreover, we divided the 40 CRC samples into low (*n* = 20) and high (*n* = 20) circCDYL2 expression groups according to the average circCDYL2 expression level. We found that p-AKT levels were higher in the high circCDYL2 group than in the low circCDYL2 group (**Figure 6E**). Consistent with this result, correlation analysis revealed a positive correlation between circCDYL2 expression and p-AKT levels (**Figure 6F**). The rescue assay also showed that enhanced CRC cell migration caused by circCDYL2 overexpression could be abolished by Ezrin knockdown (**Figures 6G, H**). These results demonstrated that circCDYL2 could inhibit CRC migration by inhibiting AKT phosphorylation *via* its binding to and upregulating Ezrin protein levels (**Figure 7**).

## Discussion

In the current study, we demonstrated for the first time the function of circCDYL2 in tumor cells. We found that circCDYL2 was upregulated in CRC tissues and cell lines and could drive CRC cell migration *in vitro*. Using RNA pulldown and RIP assays, we showed that circCDYL2 interacted with Ezrin and increased its protein levels, which promoted AKT phosphorylation.

The method we used to generate cells with high metastatic potential, based on the transwell assay, is simple and operationally convenient. Han et al. (23) also established CRC sublines using a repeated transwell assay. This research group performed five rounds of the migration assay and three rounds of the invasion assay to derive DLD1 and HCT116 CRC cell subtypes with different metastatic potentials. In our study, we performed eight rounds of the migration assay to obtain the H-RKO subline derived from the RKO cell line. This subline can be used for identifying possible metastasis-associated factors (e.g., mRNA, ncRNA, and proteins) through genomic and proteomic approaches.

Using high-throughput RNA sequencing, we identified 780 differentially expressed circRNAs in H-RKO cells compared to N-RKO cells. We found that some of these circRNAs have been reported to influence tumor metastasis. CircLMTK2 (hsa_circ_0001725) functions as a tumor promoter to enhance gastric cancer cell migration, invasion, and metastasis through the miR-150-5p/c-Myc axis (24). CircPRELID2 (hsa_circ_0006528) is upregulated in breast cancer and promotes tumor invasion and metastasis by activating the MAPK/ERK pathway through a miRNA sponge mechanism (25). CircDYNC1H1 (hsa_circ_0002060) is highly expressed in hepatocellular carcinoma and promotes cell proliferation and migration by acting as a sponge for miR-140-5p (26). CircCSPP1 (hsa_circ_0001806) also acts as a miRNA sponge in CRC (15), ovarian cancer (27), cervical cancer (28), and glioma (29) to promote proliferation and metastasis. These reports confirm the reliability of our approach and high-throughput sequencing results.

In our study, we found that the novel circRNA circCDYL2 was the most increased circRNA in H-RKO, and we proposed that it might play a key role in the initiation of migration. Its parental gene CDYL2 is overexpressed and contributes to poor prognosis in breast cancer (30). Moreover, its transcript variant CDYL2a functions as an oncogene, while CDYL2b is more likely to be a tumor suppressor in breast cancer (31). However, the roles of circCDYL2 in tumor progression remains unknown. Here, we revealed that circCDYL2 was upregulated in 40 CRC cases and four CRC cell lines. In particular, circCDYL2 expression was higher in tissues with lymphatic metastasis than without lymphatic metastasis and higher in SW620, a cell line of metastatic origin, than in SW480, which is of primary origin, suggesting that circCDYL2 could be involved in CRC metastasis. This hypothesis was supported by our functional analysis.

To investigate the possible mechanisms underlying the role of circCDYL2 in CRC migration, we first determined the subcellular localization of this circRNA and found that it mainly resided in the cytoplasm of CRC cells. As circCDYL2 is derived from an exon, our results are consistent with the generally accepted theory that exon-derived circRNAs are predominantly cytoplasmic (17). Although most published cytoplasmic circRNAs primarily function as “miRNA sponges” in tumors, circRNAs can also interact with proteins. In recent years, several protein-binding circRNAs have been shown to affect tumor progression. For instance, circ-DNMT1 can bind to both p53 and AUF1, promoting their nuclear translocation and increasing proliferation in breast cancer (18). Circ-LRIG3 interacts with EZH2 and STAT3 to facilitate EZH2-induced STAT3 phosphorylation, resulting in the malignant progression of hepatocellular carcinoma (6). Although this functional model of circRNAs is still poorly understood, we demonstrated that circCDYL2 could target the protein Ezrin.

Ezrin acts as a cross-linker between the cell membrane and the actin cytoskeleton. Ezrin is upregulated in various cancers and is associated with a poor prognosis (32, 33). Multiple studies have demonstrated that Ezrin overexpression in tumor cells increases their metastatic potential (19–22). Ezrin expression can be regulated by miRNAs (34). Moreover, Ezrin can be degraded by the ubiquitination proteasome (35). Here, we found that circCDYL2 bound to Ezrin and upregulated its protein levels, indicating that circCDYL2 might stabilize Ezrin and protect it from degradation. Besides functioning as a cytoskeletal organizer, Ezrin also plays a significant role in tumor signal transduction. It can activate the NF-κB pathway to promote the EMT in osteosarcoma (21) and CRC (22). Additionally, Ezrin can interact with AKT, which is required for AKT activation to promote breast cancer metastasis (20). The Akt pathway is a well-known pivotal oncogene pathway that regulates autophagy, angiogenesis, migration, and metastasis by activating its downstream pathways. Akt phosphorylation is indicative of Akt pathway activation. Our findings demonstrated that circCDYL2 could promote Akt phosphorylation, and this effect could be reversed by co-transfection with Ezrin siRNA. Similarly, Ezrin knockdown abrogated circCDYL2-mediated CRC cell migration. Thus, the role of circCDYL2 in CRC migration is at least in part mediated by the Ezrin/AKT axis.

In short, we identified a novel migration-related circRNA, circCDYL2, that promotes CRC migration by binding to Ezrin and promoting its upregulation to enforce AKT phosphorylation. circCDYL2 represents a potential therapeutic target for CRC.

## Data Availability Statement

The datasets presented in this study can be found in online repositories. The names of the repository/repositories and accession number(s) can be found below: https://www.ncbi.nlm.nih.gov/sra/PRJNA732514, PRJNA732514.

## Ethics Statement

The studies involving human participants were reviewed and approved by The Ethics Committee of Southern Medical University. The patients/participants provided their written informed consent to participate in this study.

## Author Contributions

XML designed the project. XML, JW, and HL performed the experiments and wrote the manuscript. XNL revised the manuscript. WL, HL, HW, YC, and QY collected the samples and interpreted part of data. All authors contributed to the article and approved the submitted version.

## Funding

This work was supported by the National Natural Science Foundation of China (Grant Numbers: 81902479, 81874074, and 82072705), Guangdong Basic and Applied Basic Research Foundation (Grant Number: 2021A1515010665), China Postdoctoral Science Foundation (Grant Number: 2019M652961), Key Scientific Research Foundation of the Wannan Medical College (Grant Number: WK2020Z01).

## Conflict of Interest

The authors declare that the research was conducted in the absence of any commercial or financial relationships that could be construed as a potential conflict of interest.

## Publisher’s Note

All claims expressed in this article are solely those of the authors and do not necessarily represent those of their affiliated organizations, or those of the publisher, the editors and the reviewers. Any product that may be evaluated in this article, or claim that may be made by its manufacturer, is not guaranteed or endorsed by the publisher.

## References

[1] Sung H Ferlay J Siegel RL Laversanne M Soerjomataram I Jemal A. Global Cancer Statistics 2020: GLOBOCAN Estimates of Incidence and Mortality Worldwide for 36 Cancers in 185 Countries. CA Cancer J Clin (2021) 71(3):209–49. 10.3322/caac.21660 33538338

[2] Wang PL Bao Y Yee MC Barrett SP Hogan GJ Olsen MN. Circular RNA Is Expressed Across the Eukaryotic Tree of Life. PloS One (2014) 9(6):e90859. 10.1371/journal.pone.0090859 24609083 PMC3946582

[3] Ma Z Shuai Y Gao X Wen X Ji J. Circular RNAs in the Tumour Microenvironment. Mol Cancer (2020) 19(1):8. 10.1186/s12943-019-1113-0 31937318 PMC6958568

[4] Li W Liu JQ Chen M Xu J Zhu D. Circular RNA in Cancer Development and Immune Regulation. J Cell Mol Med (2020). 10.1111/jcmm.16102 PMC8918416 33277969

[5] Lagunas-Rangel FA. Circular RNAs and Their Participation in Stemness of Cancer. Med Oncol (2020) 37(5):42. 10.1007/s12032-020-01373-x 32266486

[6] Sun S Gao J Zhou S Li Y Wang Y Jin L. A Novel Circular RNA Circ-LRIG3 Facilitates the Malignant Progression of Hepatocellular Carcinoma by Modulating the EZH2/STAT3 Signaling. J Exp Clin Cancer Res (2020) 39(1):252. 10.1186/s13046-020-01779-5 33222697 PMC7682056

[7] Pan Z Cai J Lin J Zhou H Peng J Liang J. A Novel Protein Encoded by Circfndc3b Inhibits Tumor Progression and EMT Through Regulating Snail in Colon Cancer. Mol Cancer (2020) 19(1):71. 10.1186/s12943-020-01179-5 32241279 PMC7114813

[8] Wu X Xiao S Zhang M Yang L Zhong J Li B. A Novel Protein Encoded by Circular SMO RNA Is Essential for Hedgehog Signaling Activation and Glioblastoma Tumorigenicity. Genome Biol (2021) 22(1):33. 10.1186/s13059-020-02250-6 33446260 PMC7807754

[9] Ren C Zhang Z Wang S Zhu W Zheng P Wang W. Circular RNA Hsa_Circ_0001178 Facilitates the Invasion and Metastasis of Colorectal Cancer Through Upregulating ZEB1 *via* Sponging Multiple miRNAs. Biol Chem (2020) 401(4):487–96. 10.1515/hsz-2019-0350 31747371

[10] Chen RX Chen X Xia LP Zhang JX Pan ZZ Ma XD. N(6)-Methyladenosine Modification of Circnsun2 Facilitates Cytoplasmic Export and Stabilizes HMGA2 to Promote Colorectal Liver Metastasis. Nat Commun (2019) 10(1):4695. 10.1038/s41467-019-12651-2 31619685 PMC6795808

[11] Chen LY Zhi Z Wang L Zhao YY Deng M Liu YH. NSD2 Circular RNA Promotes Metastasis of Colorectal Cancer by Targeting miR-199b-5p-Mediated DDR1 and JAG1 Signalling. J Pathol (2019) 248(1):103–15. 10.1002/path.5238 30666650

[12] Li X Wang J Zhang C Lin C Zhang J Zhang W. Circular RNA Circitga7 Inhibits Colorectal Cancer Growth and Metastasis by Modulating the Ras Pathway and Upregulating Transcription of Its Host Gene ITGA7. J Pathol (2018) 246(2):166–79. 10.1002/path.5125 29943828

[13] Schmittgen TD Livak KJ. Analyzing Real-Time PCR Data by the Comparative C(T) Method. Nat Protoc (2008) 3(6):1101–8. 10.1038/nprot.2008.73 18546601

[14] Paschalis A Sheehan B Riisnaes R Rodrigues DN Gurel B Bertan C. Prostate-Specific Membrane Antigen Heterogeneity and DNA Repair Defects in Prostate Cancer. Eur Urol (2019) 76(4):469–78. 10.1016/j.eururo.2019.06.030 PMC6853166 31345636

[15] Wang Q Shi L Shi K Yuan B Cao G Kong C. CircCSPP1 Functions as a ceRNA to Promote Colorectal Carcinoma Cell EMT and Liver Metastasis by Upregulating Col1a1. Front Oncol (2020) 10:850. 10.3389/fonc.2020.00850 32612946 PMC7308451

[16] Glažar P Papavasileiou P Rajewsky N. Circbase: A Database for Circular RNAs. Rna (2014) 20(11):1666–70. 10.1261/rna.043687.113 PMC4201819 25234927

[17] Long F Lin Z Li L Ma M Lu Z Jing L. Comprehensive Landscape and Future Perspectives of Circular RNAs in Colorectal Cancer. Mol Cancer (2021) 20(1):26. 10.1186/s12943-021-01318-6 33536039 PMC7856739

[18] Du WW Yang W Li X Awan FM Yang Z Fang L. A Circular RNA Circ-DNMT1 Enhances Breast Cancer Progression by Activating Autophagy. Oncogene (2018) 37(44):5829–42. 10.1038/s41388-018-0369-y 29973691

[19] Xi M Tang W. Knockdown of Ezrin Inhibited Migration and Invasion of Cervical Cancer Cells *In Vitro* . Int J Immunopathol Pharmacol (2020) 34:2058738420930899. 10.1177/2058738420930899 32674647 PMC7370327

[20] Li N Kong J Lin Z Yang Y Jin T Xu M. Ezrin Promotes Breast Cancer Progression by Modulating AKT Signals. Br J Cancer (2019) 120(7):703–13. 10.1038/s41416-019-0383-z PMC6461860 30804430

[21] Liu P Yang P Zhang Z Liu M Hu S. Ezrin/NF-κb Pathway Regulates EGF-Induced Epithelial-Mesenchymal Transition (EMT), Metastasis, and Progression of Osteosarcoma. Med Sci Monit (2018) 24:2098–108. 10.12659/msm.906945 PMC5907830 29628496

[22] Li Y Lin Z Chen B Chen S Jiang Z Zhou T. Ezrin/NF-kB Activation Regulates Epithelial- Mesenchymal Transition Induced by EGF and Promotes Metastasis of Colorectal Cancer. BioMed Pharmacother (2017) 92:140–48. 10.1016/j.biopha.2017.05.058 28535417

[23] Han K Wang FW Cao CH Ling H Chen JW Chen RX. CircLONP2 Enhances Colorectal Carcinoma Invasion and Metastasis Through Modulating the Maturation and Exosomal Dissemination of microRNA-17. Mol Cancer (2020) 19(1):60. 10.1186/s12943-020-01184-8 32188489 PMC7079398

[24] Wang S Tang D Wang W Yang Y Wu X Wang L. Circlmtk2 Acts as a Sponge of miR-150-5p and Promotes Proliferation and Metastasis in Gastric Cancer. Mol Cancer (2019) 18(1):162. 10.1186/s12943-019-1081-4 31722712 PMC6854648

[25] Gao D Qi X Zhang X Fang K Guo Z Li L. Hsa_circRNA_0006528 as a Competing Endogenous RNA Promotes Human Breast Cancer Progression by Sponging miR-7-5p and Activating the MAPK/ERK Signaling Pathway. Mol Carcinog (2019) 58(4):554–64. 10.1002/mc.22950 30520151

[26] Wang ZY Zhu Z Wang HF Qin B Liu J Yao XH. Downregulation of Circdync1h1 Exhibits Inhibitor Effect on Cell Proliferation and Migration in Hepatocellular Carcinoma Through miR-140-5p. J Cell Physiol (2019) 234(10):17775–85. 10.1002/jcp.28403 30864145

[27] Li QH Liu Y Chen S Zong ZH Du YP Sheng XJ. Circ-CSPP1 Promotes Proliferation, Invasion and Migration of Ovarian Cancer Cells by Acting as a miR-1236-3p Sponge. BioMed Pharmacother (2019) 114:108832. 10.1016/j.biopha.2019.108832 30965236

[28] Yang W Xie T. Hsa_circ_CSPP1/MiR-361-5p/ITGB1 Regulates Proliferation and Migration of Cervical Cancer (CC) by Modulating the PI3K-Akt Signaling Pathway. Reprod Sci (2020) 27(1):132–44. 10.1007/s43032-019-00008-5 32046405

[29] Xue YF Li M Li W Lin Q Yu BX Zhu QB. Roles of Circ-CSPP1 on the Proliferation and Metastasis of Glioma Cancer. Eur Rev Med Pharmacol Sci (2020) 24(10):5519–25. 10.26355/eurrev_202005_21337 32495924

[30] Siouda M Dujardin AD Barbollat-Boutrand L Mendoza-Parra MA Gibert B Ouzounova M. CDYL2 Epigenetically Regulates MIR124 to Control NF-κb/STAT3-Dependent Breast Cancer Cell Plasticity. iScience (2020) 23(6):101141. 10.1016/j.isci.2020.101141 32450513 PMC7251929

[31] Yang LF Yang F Zhang FL Xie YF Hu ZX Huang SL. Discrete Functional and Mechanistic Roles of Chromodomain Y-Like 2 (CDYL2) Transcript Variants in Breast Cancer Growth and Metastasis. Theranostics (2020) 10(12):5242–58. 10.7150/thno.43744 PMC7196301 32373210

[32] Zhang R Zhang S Xing R Zhang Q. High Expression of EZR (Ezrin) Gene is Correlated With the Poor Overall Survival of Breast Cancer Patients. Thorac Cancer (2019) 10(10):1953–61. 10.1111/1759-7714.13174 PMC6775014 31452341

[33] Piao J Liu S Xu Y Wang C Lin Z Qin Y. Ezrin Protein Overexpression Predicts the Poor Prognosis of Pancreatic Ductal Adenocarcinomas. Exp Mol Pathol (2015) 98(1):1–6. 10.1016/j.yexmp.2014.11.003 25445504

[34] Yan H Sun BM Zhang YY Li YJ Huang CX Feng FZ. Upregulation of miR-183-5p Is Responsible for the Promotion of Apoptosis and Inhibition of the Epithelial-Mesenchymal Transition, Proliferation, Invasion and Migration of Human Endometrial Cancer Cells by Downregulating Ezrin. Int J Mol Med (2018) 42(5):2469–80. 10.3892/ijmm.2018.3853 PMC6192766 30226564

[35] Yuan YP Zhao H Peng LQ Li ZF Liu S Yuan CY. The SGK3-Triggered Ubiquitin-Proteasome Degradation of Podocalyxin (PC) and Ezrin in Podocytes Was Associated With the Stability of the PC/ezrin Complex. Cell Death Dis (2018) 9(11):1114. 10.1038/s41419-018-1161-1 30385740 PMC6212497

