# Supplementary material for: circCDYL2, Overexpressed in Highly Migratory Colorectal Cancer Cells, Promotes Migration by Binding to Ezrin
Source: Front Oncol. 2021 Aug 17;11:716073. doi: 10.3389/fonc.2021.716073 (PMC8416158; doi:10.3389/fonc.2021.716073)
Supplement: Supplementary file 2 [file Table_1.doc]

**Supplementary Table 1. Primer sequences are used in PCR or RT- qPCR**

|  | | Forward primer (5’-3’) | Reverse primer (5’-3’) | |
| --- | --- | --- | --- | --- |
| hsa_circ_0003331 | GGGGCCTACCGAAAGATC | | GGCAAGGGCATTACCACA |  |
| hsa_circ_0002972 | TCCCATCTACCCTCCACC | | CTTCTTCCCAGCCTTTGC |  |
| hsa_circ_0008939 | ATTGCCCTGGACTTCCTA | | ATGTGCTTGCGGACCTTC |  |
| hsa_circ_0004087  (circCDYL2) | GAATCACGCTACACTGGC | | GGACTGCTTCCCTGACTTG |  |
| hsa_circ_0000024 | ACTCGCCTTTGTGAACCT | | GGTAGCCCAGGAAGGAAT |  |
| hsa_circ_0001264 | AAGGAAAGCCTCAGAAGT | | AAGAAGCAGGAGATTTGG |  |
| hsa_circ_0002060 | CCCGCCTTGACCAGATGA | | CCGTGGCAGACAGCAAAT |  |
| CDYL2 | CTTATCCGATGGAAAGGCTACG | | GCTTCCCTGACTTGATCCTCTT |  |
| GAPDH | GGAGCGAGATCCCTCCAAAAT | | GGCTGTTGTCATACTTCTCATGG |  |
| U6 | CTCGCTTCGGCAGCACA | | AACGCTTCACGAATTTGCGT |  |
