# Supplementary material for: circCDYL2, Overexpressed in Highly Migratory Colorectal Cancer Cells, Promotes Migration by Binding to Ezrin
Source: Front Oncol. 2021 Aug 17;11:716073. doi: 10.3389/fonc.2021.716073 (PMC8416158; doi:10.3389/fonc.2021.716073)
Supplement: Supplementary file 3 [file Table_2.doc]

**Supplementary Table 2. siRNAs are used for knockdown of circCDYL2 or Ezrin**

|  | Target sequences (5’-3’) |
| --- | --- |
| circCDYL2 siRNA-1 | CTCGGTTGAAAGGATTGTA |
| circCDYL2 siRNA-2 | AGAACGGGCTCGGTTGAAA |
| Ezrin siRNA | CTGCGGAGCTTGCAGAATA |
